# Efficient expression vectors and host strain for the production of recombinant proteins by *Yarrowia lipolytica* in process conditions

**DOI:** 10.1186/s12934-019-1218-6

**Published:** 2019-10-10

**Authors:** Young-Kyoung Park, Marie Vandermies, Paul Soudier, Samuel Telek, Stéphane Thomas, Jean-Marc Nicaud, Patrick Fickers

**Affiliations:** 1 Micalis Institute, INRA, AgroParisTech, Université Paris-Saclay, 78350 Jouy-en-Josas, France; 2 Microbial Processes and Interactions, TERRA Teaching and Research Centre, University of Liège - Gembloux Agro-Bio Tech, Gembloux, Belgium; 3 Micalis Institute, UMR1319, Team BIMLip: Integrative Metabolism of Microbial Lipids, INRA-AgroParisTech, Domaine de Vilvert, 78352 Jouy-en-Josas, France

**Keywords:** Promoter, Regulation, Induction, Synthetic promoter, Erythritol, Protein secretion, Upstream activating sequence, *Yarrowia lipolytica*, CalB

## Abstract

**Background:**

The oleaginous yeast *Yarrowia lipolytica* is increasingly used as an alternative cell factory for the production of recombinant proteins. Recently, regulated promoters from genes *EYK1* and *EYD1*, encoding an erythrulose kinase and an erythritol dehydrogenase, respectively, have been identified and characterized in this yeast. Hybrid promoters up-regulated by polyols such as erythritol and erythrulose have been developed based on tandem copies of upstream activating sequences from *EYK1* (UAS1_EYK1_) and *XPR2* (encoding extracellular protease, UAS1_XPR2_) promoters.

**Results:**

The strength of native (p*EYD1*) and engineered promoters (*pEYK1*-*3AB* and *pHU8EYK*) was compared using the extracellular lipase CalB from *Candida antarctica* as a model protein and a novel dedicated host strain. This latter is engineered in polyol metabolism and allows targeted chromosomal integration. In process conditions, engineered promoters *pEYK1*-*3AB* and *pHU8EYK* yielded 2.8 and 2.5-fold higher protein productivity, respectively, as compared to the reference *pTEF* promoter. We also demonstrated the possibility of multicopy integration in the newly developed host strain. In batch bioreactor, the CalB multi-copy strain RIY406 led to a 1.6 fold increased lipase productivity (45,125 U mL^−1^) within 24 h as compared to the mono-copy strain.

**Conclusions:**

The expression system described herein appears promising for recombinant extracellular protein production in *Y. lipolytica*.

## Background

The oleaginous yeast *Yarrowia lipolytica* is increasingly used as an alternative to model yeasts such as *Saccharomyces cerevisiae* or *Komagataella phaffii* (*Pichia pastoris*). for the production of recombinant proteins [1, 2]. More than one hundred heterologous proteins have already been produced at high yield, highlighting its potential utilization as a cell factory. *Y. lipolytica* has been also shown able to produce efficiently chemicals such as citric acid [3], itaconic acid [4], erythritol [5], erythrulose [6] and lipid for biodiesel and biojet fuel [7, 8]. In nature, *Y. lipolytica* can grow in environments rich in lipids and proteins due to its ability to synthesize and secrete hydrolytic enzymes such as proteases or lipases [9, 10]. Based on these metabolic features, several engineering tools such as strong promoters and efficient sorting signals, including the prepro sequence of the *LIP2* gene encoding an extracellular lipase, have been developed [1, 11]. The promoter from the *XPR2* gene encoding alkaline extracellular protease was the first developed for recombinant gene expression [12]. Its functional dissection led to the identification of an upstream activating sequence (UAS1_XPR2_), later used to construct constitutive hybrid promoters [13]. These are based on UAS1_XPR2_ repeats fused upstream of the minimal *LEU2* promoter (*mLEU2*) [14, 15]. The number of UAS1_XPR2_ repeats was found to modulate the strength of the promoter, allowing thus a fine-tuning of gene expression. A similar strategy was also applied to design constitutive promoters derived from the *TEF1* gene encoding the translation elongation factor 1α [16]. Regulated promoters derived from the *LIP2* and *POX2* genes encoding an extracellular lipase and an acyl-CoA oxidase, respectively, have been also developed for recombinant gene expression [17]. Although these regulated promoters delivered strong gene expression, their utilization at industrial scale is impeded by the hydrophobic nature of their inducers (i.e. triglycerides and fatty acids).

Recently, we have characterized two genes, namely *EYD1* and *EYK1*, involved in erythritol catabolic pathway [6, 18]. Gene *EYD1* was found to encode an erythritol dehydrogenase able to convert erythritol to erythrulose, while gene *EYK1* was suggested to encode an erythrulose kinase. Induction of *EYK1* native promoter (*pEYK300*) is significantly increased, in a dose-dependent manner, in the presence of erythritol or erythrulose, and drastically reduced in the presence of glycerol and glucose. Moreover, we demonstrated that erythrulose is a better inducer than erythritol [19]. The dissection of the *EYK1* promoter highlighted the existence of two UAS, namely UAS1_EYK1_ and UAS2_EYK1_. Using a reporter system based on yellow fluorescent protein (YFP) and mutated promoter, UAS1_EYK1_ was identified as essential for promoter induction by both erythritol and erythrulose. By contrast, UAS2_EYK1_ was found involved in repression by glucose. Synthetic promoters were constructed by addition of multiple copies of UAS1_EYK1_ or UAS1_XPR2_ upstream of the native *pEYK300* promoter. These promoters yielded, respectively, 3.2 and 15.6-fold higher expression levels of YFP-encoding gene than those obtained with wild-type *pEYK300* promoter [19]. Carly et al. [6, 18] found that the disruption of the *EYK1* gene impairs the ability of yeast cells to fully metabolize erythritol, since an *eyk1Δ* mutant could only convert erythritol into erythrulose. Therefore, with such a mutant, erythritol and/or erythrulose could be used as a free inducer as demonstrated previously [19]. Promoter engineering efforts were pursued with a reporter system based on RedStar fluorescent protein [20]. It concerned the influence of UAS1_EYK1_ repeats and core element (*EYK1*, *TEF*) on promoter strength in wild type and *eyk1Δ* mutant.

Based on previous results, three promoters, namely *pEYK1*-*3AB* which comprises three repeats of UAS1_EYK1_, *pHU8EYK* which comprises eight copies UAS1_XPR2_ from *XPR2* gene and the native promoter of gene *EYD1* seemed to be promising to drive the production of recombinant proteins [19, 20]. Herein, these promoters were challenged for the production in process conditions of a protein of industrial interest, the lipase CalB from *Candida antarctica.* For that purpose, the pro-CalB nucleic acid sequence was codon-optimized for *Y. lipolytica*, fused with the secretion signal from the *LIP2* gene, and cloned under the control of promoters *pEYD1*, *pEYK1*-*3AB*, *pHU8EYK*, and of strong constitutive promoter *pTEF* used for comparison. The different CalB expression cassettes were then introduced in a novel host strain specifically developed for erythritol/erythrulose-inducible expression systems. For the different constructed strains, CalB gene expression and extracellular activity were monitored in process conditions, during cultures in bioreactor.

## Methods

### Media and culture conditions

*Escherichia coli* strains were grown at 37 °C in Luria–Bertani medium supplemented with kanamycin sulfate (50 µg mL^−1^). *Y. lipolytica* strains were grown at 28 °C in YPD medium or in YNB medium (1.7 g L^−1^ yeast nitrogen base without amino acids and ammonium sulfate, YNBww (BD Difco, Franklin Lakes, NJ, USA), 50 mM phosphate buffer pH 6.8 supplemented with carbon and nitrogen sources, as described in Barth and Gaillardin [21]. For YNBD medium, glucose 10 g L^−1^ and NH_4_Cl 5 g L^−1^ were added to YNB medium. For YNBE medium, erythritol 10 g L^−1^ and NH_4_Cl 5 g L^−1^ were added to YNB medium. For YNBGE medium, glycerol 10 g L^−1^, erythritol 10 g L^−1^, yeast extract 5 g L^−1^ (Yeast extract UF, BD Difco), soytone 5 g L^−1^ (Select soytone, BD Difco) were added to YNB medium. YNBG_2_E medium is the same as YNBGE except it contained glycerol 20 g L^−1^. Medium contained lysine (0.08%, w/v) and/or uracil (0.01%, w/v) to meet auxotrophic requirement. Hygromycin (200 µg mL^−1^) was added for transformant selection. Solid media contained agar 1.5 % (w/v). Phosphate buffered saline (PBS) contained NaCl 8 g L^−1^, KCl 0.2 g L^−1^, Na_2_HPO_4_ 1.44 g L^−1^ and KH_2_PO4 0.24 g L^−1^.

Cultures in bioreactor were inoculated at an initial optical density at 600 nm (OD_600_) of 0.5 with PBS washed cells from a 16-h preculture in YPD medium. Cultures in 2Mag bioREACTOR (Munich, Germany) were performed in triplicate for 48 h in 10 mL of YNBGE medium with agitation set at 800 rpm. Cultures in DASGIP bioreactor (DASbox Mini Bioreactors SR0250ODLS, Eppendorf, Hamburg, Germany) were performed in duplicate for 48 h in 150 mL of YNBG_2_E medium supplemented with 500 µL L^−1^ antifoam (Tego KS911, Evonik, Essen, Germany). Airflow was set at 1 vvm, agitation was ranged from 800 to 950 rpm to ensure a dissolved oxygen level above 20% and pH was automatically adjusted to 6.8 by addition of H_3_PO_4_ 8 M or NaOH 12.5 M. Culture in Duetz deepwell plate (24-well plate with pyramidal bottom, Kühner AG, Birsfelden, Switzerland) were performed in 2 mL of YNBG_2_E medium for 48 h; they were inoculated with of 200 μL of a 24 h precultures carried out in 400 µL YPD in 48-well microplates (multiwell cell culture plates, flat bottom, TC-treated, VWR, Radnor, PA, USA) for 24 h.

### Strains and plasmids construction

Standard molecular genetic techniques were used in this study [22]. Restriction enzymes were obtained from New England Biolabs (MA, USA). PCR amplifications were performed in an Applied Biosystems 2720 thermal cycler with GoTaq DNA polymerases (Promega, WI, USA) or Q5 High-Fidelity DNA Polymerase (New England Biolabs). PCR fragments were purified with a QIAgen Purification kit (Qiagen, Hilden, Germany) and plasmids DNA were isolated with a QIAprep Spin Miniprep kit (Qiagen). All strains and plasmids used in this study are listed in Table 1.

**Table 1:** Strains and plasmids used in this study

Strain (plasmid)	Genotype	References
*E. coli*		
JME547 (JMP547)	pUB4-Cre-Hyg	[23]
JME1046 (JMP1046)	JMP62-*URA3ex*-*pTEF*	[34]
JME3267 (JMP3267)	PUT *lys5*::*URA3ex*	This work
JME3739 (JMP3739)	JMP62-*URA3ex*-*pTEF*-CalB	This work
RIE124 (RIP124)	PUT *eyk1*::*URA3ex*	[33]
JME4001 (JMP4001)	JMP62-URA3ex-pHU8EYK-YFP	Unpublished
JME4123 (JMP4123)	pUC57-*pEYK1*-*3AB*	[19]
JME4230 (JMP4230)	JMP62-*URA3ex*-*pHU8EYK*	This work
JME4243 (JMP4243)	JMP62-*URA3ex*-*pHU8EYK* CalB	This work
RIE132 (RIP132)	pGEMT-easy-Cre-*EYK1*	[33]
JME4266 (JMP4266)	JMP62-*URA3ex*-*pEYK1*-*3AB*	This work
JME4365 (JMP4365)	JMP62-*URA3ex*-*pEYK1*-*3AB*-CalB	This work
JME4579 (JMP4579)	JMP62-*LYS5ex*-*pEYK1*-*3AB*-CalB	This work
JME4590 (JMP4590)	JMP62-*URA3ex*-*pEYD1*-CalB	This work
RIE279 (RIP279)	JMP62-*LYS5ex*	This work
*Y. lipolytica*		
JMY1212	*MATA ura3*-*302 leu2*-*270*-*LEU2*-*Zeta*, *xpr2*-*322*, *lip2Δ*, *lip7Δ*, *lip8Δ*	[30]
JMY5207	*MATA ura3*-*302 leu2*-*270*-*LEU2*-*Zeta*, *xpr2*-*322*, *lip2Δ*, *lip7Δ*, *lip8Δ*, *lys5::URA3ex*	This work
JMY7121	*MATA ura3*-*302 leu2*-*270*-*LEU2*-*Zeta*, *xpr2*-*322*, *lip2Δ*, *lip7Δ*, *lip8Δ*, *lys5Δ*	This work
JMY7123	*MATA ura3*-*302 leu2*-*270*-*LEU2*-*Zeta*, *xpr2*-*322*, *lip2Δ*, *lip7Δ*, *lip8Δ*, *lys5Δ*, *eyk1::URA3ex*	This work
JMY7126	*MATA ura3*-*302 leu2*-*270*-*LEU2*-*Zeta*, *xpr2*-*322*, *lip2Δ*, *lip7Δ*, *lip8Δ*, *lys5Δ*, *eyk1Δ*	This work
JMY7536	JMY7126 + *pTEF*-CalB-*URA3ex*	This work
JMY7539	JMY7126 + *pEYK1*-*3AB*-CalB-*URA3ex*	This work
JMY7544	JMY7126 + *pHU8EYK*-CalB-*URA3ex*	This work
JMY7548	JMY7126 + *pEYD1*-CalB-*URA3ex*	This work
RIY368	JMY7539 + *LYS5ex*	This work
RIY394	JMY7536 + *LYS5ex*	This work
RIY406	JMY7126 + *pEYK1*-*3AB*-CalB-*URA3ex *+ *pEYK1*-*3AB*-CalB-*LYS5ex*	This work

**Fig. 1:**
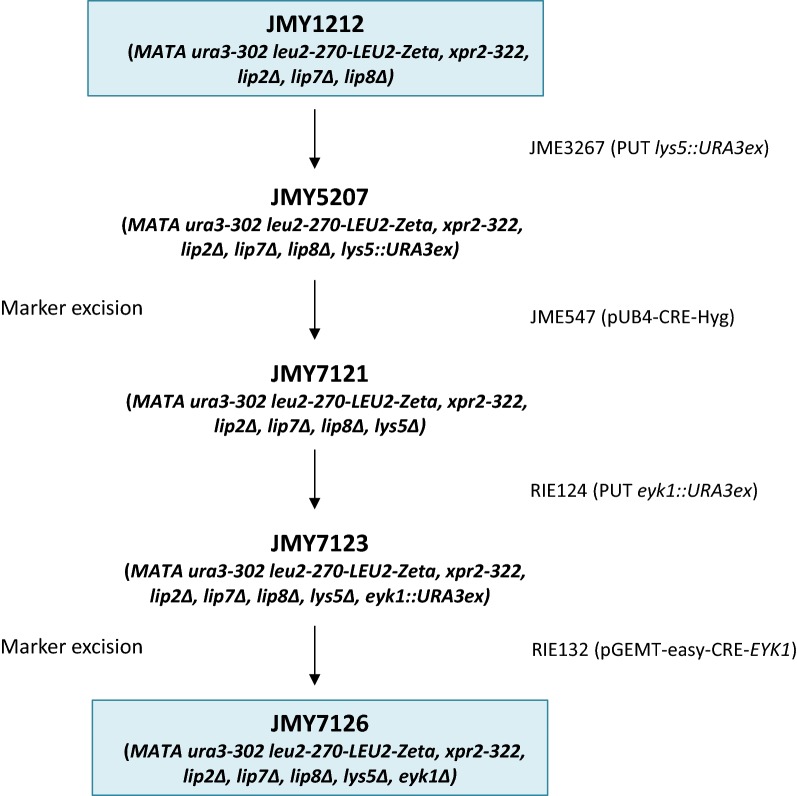
Construction of *Y. lipolytica* strain JMY7126. The auxotrophic strain JMY1212 [27] was used as parental strain. *LYS5* and *EYK1* genes were successively disrupted with the corresponding purified disruption cassette obtained from JMP3267 and RIP124 by *Not*I digestion, respectively. *URA3ex* auxotrophy marker was excised from strains using Cre-Hyg or Cre-*EYK1* replicative plasmids (JME547 and RIE132, respectively) as described in [23, 33]

For the construction of JMY7126 (Fig. 1), *LYS5* and *EYK1* were disrupted using PUT cassettes according to Fickers et al. [23]. For *LYS5* disruption, the promoter (P) and terminator (T) regions of the gene were amplified by PCR with primer pairs LYS5-P1/LYS5-P2 and LYS5-T1/LYS5-T2, respectively. Primers LYS5-P1 and LYS5-T2, contained sequence of *Not*I restriction site while primers LYS5-P2 and LYS5-T1 contained the sequence of the I-*Sce*I restriction (Table 2). The corresponding amplicons were purified and used as a template for the second PCR step resulting PT fragment, that was subsequently cloned to pCR4Blunt-TOPO plasmid after purification (Invitrogen, CA, USA). Finally, *URA3ex* marker from JMP1046 was introduced at the I-*Sce*I site of this plasmid, yielding plasmid JMP3267. The *LYS5* PUT cassette obtained by *Not*I digestion of JMP3267 was used to transform *Y. lipolytica* strain JMY1212 to yield JMY5207 (Fig. 1). The disruption of *LYS5* was verified by auxotrophy check on YNBD and YNBD-lysine. In that strain, *URA3ex* marker was rescued by transient expression of Cre recombinase using the replicative plasmid JMP547 as described previously [23]. In the resulting strain JMY7121, *EYK1* was disrupted using a PUT cassette obtained from plasmid RIE124 by *Not*I digestion. The disruption of *EYK1* was verified by colony PCR with primers pair preTEYK Fw/postPEYK Rv and growth on YNBD-lysine and YNBE-lysine. This yielded to strain JMY7123 that was further transformed with plasmid RIE132 in order to excise the *URA3ex* marker. This yielded to the final strain JMY7126 (Table 1, Fig. 1). The loss of the replicative plasmid was checked by replica plating on YPD supplemented or not with hygromycin for JME547, or on YNBD and YNBE for RIE132. To restore *LYS5* prototrophy, strains were transformed with the expression cassette of plasmid RIE279 obtained by *Not*I digestion, and gene integration was verified by colony PCR using primer LPR-R and LYS5PR.

**Table 2:** Primers used in this study

Primers	Sequence (5′ to 3′)	Restriction site/utilisation
LYS5-P1	ATAAGAAT**GCGGCCGC**CGACTAAATTTCGACCCAC	*Not*I, *LYS5* disruption
LYS5-P2	CG**ATTACCCTGTTATCCCTA**GCGTAACTCGCTACTAGGCCGCCACC	I-*Sce*I, *LYS5* disruption
LYS5-T1	CG**TAGGGATAACAGGGTAAT**ATAGCGTAACTATAACGGTCCTAAGGTAGCGAAGGCGTTGGTGCTCTCTCGGAAGTAG	I-*Sce*I, *LYS5* disruption
LYS5-T2	ATAGTTTA**GCGGCCGC**AAAAATGTCCGCCATTGAGTGTTG	*Not*I, *LYS5* disruption
LPR-R	GCTAGATAGAGTCGAGAATTACCCTG	*LYS5* prototrophy
LYS5PR	TCGGTGCGTGTGAAAGACAC	*LYS5* prototrophy
preTEYK Fw	GTGTTTGACATTTTGTTTTGTGTGAGT	*Verification of EYK1 disruption*
postPEYK Rv	TACACACTCACACTCACCAGAACATC	*Verification of EYK1 disruption*
ClaI-pEYD1-Fw	CCCATCGATGGAAACCTTAATAGGAGACTACTTCC	*pEYD1* cloning
no AvrII-pEYD1-Fw	CCTCGTGTCCGGGCTAGGGCAGAAACAGCTC	*pEYD1* cloning, *pEYD1* verification
no AvrII-pEYD1-Rev	GAGCTGTTTCTGCCCTAGCCCGGACACGAGG	*pEYD1* cloning
BamHI-pEYD1-Rev	TGTGTATGTGTGTGTGTGTGTGTGTGTGTGTTTG	*pEYD1* cloning
pTEF-internal-Fw	TCTGGAATCTACGCTTGTTCA	*pTEF* verification
EYK300-Fw	GCATCTACTTTTCTCTATACTGTACGTTTCAATCTGGG	*pEYK1*-*3AB*, *pHU8EYK* verification
CalB-prepro-Fw	ATGAAGCTGCTGTCTCTGACC	CalB verification
CalB-internal-Rev1	CCACCTTAGATCGAATAGAAGGG	CalB verification
CalB-Rev	TTAAGGGGTGACAATACCAGAAC	CalB verification
ACT-F	TCCAGGCCGTCCTCTCCC	qPCR
ACT-R	GGCCAGCCATATCGAGTCGCA	qPCR
CalB-internal-Fw	TCTCTGCTCCTTCTGTGTGG	qPCR
CalB-internal-Rev2	GTCGAACAGAGGTCCACAGA	qPCR

**Fig. 2:**
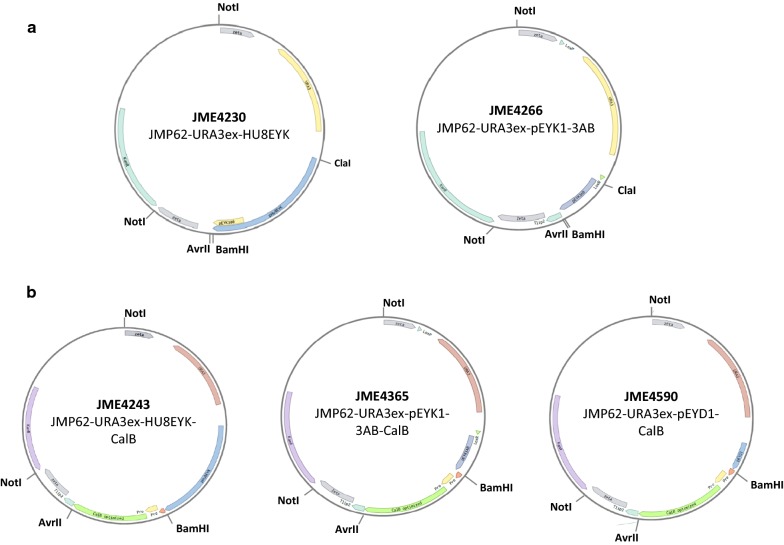
Maps of expression plasmids. **a** The erythritol inducible plasmids JME4230 containing *pHU8EYK* and JME4266 containing *pEYK1*-*3AB*. **b** CalB expressing plasmids JME4243 (*pHU8EYK*), JME4365 (*pEYK1*-*3AB*), and JME4590 (*pEYD1*)

The erythritol-inducible plasmids were constructed from JMP1046 by replacing the *pTEF* by inducible promoters (namely *pEYK1*-*3AB*, *pHU8EYK1*, plasmid JMP4123 and JMP4001, respectively) by digestion with *Cla*I and *Bam*HI and subsequent ligation. Here, *pEYK300A3B* described in Trassaert et al. [19] was renamed *pEYK1*-*3AB* according to Park et al. [20]. The *Y. lipolytica LIP2 pre*-*CalB pro*-*CalB* gene was codon-optimized by Biocatalysts LTD, (Cardiff, UK) and synthesized by Geneart (Regensburg, Germany). The sequence of the optimized synthetic gene (15ACCYPP_1762989_LIP2-CalB-Yl-Opt) is displayed in Additional file 1: Table S1. LIP2*pre*-*CalBpro*-*CalB* gene (CalB) was cloned into the vectors JME1046 (p*TEF*), JME4266 (p*EYK1*-*3AB*) and JME4230 (p*HU8EYK*) at *BamH*I/*Avr*II restriction sites (Fig. 2 and Table 1). To obtain a p*EYD1*-CalB construct, promoter *pEYK1*-*3AB* from plasmid JMP4365 was exchanged by p*EYD1* obtained by PCR on genomic DNA of *Y. lipolytica* using primer ClaI-pEYD1-Fw and BamHI-pEYD1-Rev and subsequent enzyme digestion with *Cla*I and *Bam*HI. For the construction of plasmid JME4579, the *URA3*ex marker was exchanged with the *LYS5*ex marker by I-*Sce*I digestion and ligation. Gene expression cassettes were obtained by *Not*I digestion of the corresponding plasmid and used to transform *Y. lipolytica* strains JMY7126 by the lithium acetate method as described previously [24].

### Analytical methods

#### Cell growth monitoring

Cell growth was monitored by optical density at 600 nm (OD_600_). 1 unit of OD_600_ corresponds to 0.29 g of dry cell weight (DCW).

#### Quantification of CalB gene expression

RNA extraction and cDNA synthesis were performed as described previously [17] on 2.2 × 10^7^ cells of strains JMY7536, JMY7539, JMY7544, and JMY7548 after 24 h of culture in 2Mag bioREACTOR. qPCR was performed with primer pairs CalB-internal-Fw/CalB-internal-R2 and ACT-F/ACT-R (Table 2) for CalB and actin genes, respectively. CalB gene expression levels were standardized relative to the expression level of the actin gene [17]. Experiments were performed in triplicate.

#### Lipase activity

Lipase activity in culture supernatants was determined by monitoring the hydrolysis of para-nitrophenyl butyrate (p-NPB), according to Fickers et al. [25]. p-NPB dissolved in acetonitrile (20% v/v) was added to a final concentration of 1 mM into 100 mM phosphate buffer, pH 7.2, containing 100 mM NaCl. The resulting solution was sonicated for 2 min on ice. The reaction was initiated by addition of 20 µL of culture supernatant to 1 mL of p-NPB solution. The release of para-nitrophenol (P-NP) was monitored for 3 min at 405 nm (A_405_), considering the molar extinction coefficient of P-NP (εPNP) equal to 0.0148 µM^−1^ cm^−1^. When necessary, supernatant samples were diluted to obtain initial velocities below OD_405_ of 0.3 U min^−1^. All lipase activity assays were performed at least in duplicate from two independent cultures. One unit of lipase activity was defined as the amount of enzyme releasing 1 µmol p-NPB per minute at 25 °C and pH 7.2 (U mL^−1^). Specific lipase activity was defined as lipase activity per gram of dry cell weight (U gDCW^−1^). Lipase volumetric production rate was defined as lipase activity per hour of culture (U mL^−1^ h^−1^), while lipase specific production rate was defined as unit of lipase activity per gram of DCW and per hour (U gDCW^−1^ h^−1^).

#### Protein electrophoresis

Proteins were subjected to sodium dodecyl sulfate (SDS)-polyacrylamide gel electrophoresis (PAGE) on a Novex™ 12% Tris–Glycine Mini Gel (Thermo Fisher Scientific), according to Laemmli [26]. Four milliliter of prestained Protein Marker IV (AppliChem GmbH, Darmstadt, Germany) were used as molecular weight standards, and a supernatant sample contained 40 U of lipase in a volume 5 µL.

#### Carbon source concentration

Polyol concentrations in culture supernatants were determined by HPLC (Agilent 1200 series, Agilent Technologies, Santa Clara, CA, USA). Compounds were eluted from an Aminex HPX-87H column (300 × 7.8 mm, BioRad, Hercules, CA, USA) at 65 °C, using a mobile phase consisting of 5 mM H_2_SO_4_ solution at a flow rate of 0.5 mL/min. Glycerol and erythritol were detected using refractive index and erythrulose was detected using UV absorption at 210 nm. Specific glycerol uptake rate was defined as gram of glycerol per gram of DCW and hour (g gDCW^−1^ h^−1^).

## Results and discussion

### Construction of the host strain JMY7126 for erythritol-inducible based expression system

*Yarrowia lipolytica* strain JMY1212 was previously developed to target integration of an expression cassette at a zeta docking platform located at the *LEU2* locus [27]. With such a strain, any variability of recombinant gene expression related to the integration locus could be avoided, which is a prerequisite for this study. Moreover, such a feature leads to precisely genetically characterized recombinant strains, which is of importance when developing an industrial chassis. Strain JMY1212 is also disrupted for gene *XPR2* encoding AEP alkaline protease and for *LIP2*, *LIP7* and *LIP8* encoding the main extracellular lipases in *Y. lipolytica* [10, 28]. Despite strain JMY1212 has been used successfully for heterologous gene expression [27, 29–31], it suffers from several drawbacks: (i) it possesses only a single auxotrophy based on uracil metabolism (*ura3*), impairing thus multiple genome editions; (ii) it is able to metabolize erythritol and erythrulose, preventing thus their utilization as free inducer. To adapt the potentialities of JMY1212 to the requirements of a versatile, erythritol/erythrulose-inducible expression system, the strain was further genetically engineered. Firstly, gene *LYS5* encoding saccharopine dehydrogenase [32] was disrupted with the *URA3ex* cassette. The resulting lysine auxotroph strain was named JMY5207 (Fig. 1).

In order to use erythritol as a free inducer, it is requested to dispose of a host strain unable to metabolize this polyol. We demonstrated in a previous study that erythrulose, the first intermediate of the erythritol catabolic pathway [18] is a better inducer than erythritol [19]. Therefore, the second step of JMY1212 strain improvement was to disrupt gene *EYK1.* Practically, the gene was disrupted in strain JMY7121, an auxothroph derivative of JMY5207 (Fig. 1) using a PUT cassette obtained from RIP124 by *Not*I digestion. This yielded finally to strain JMY7126, after *URA3ex* marker excision in strain JMY7123. With such a strain, we obtained a useful system to generate autonomously erythrulose from low-cost erythritol, and allowing the use of these polyols as free inducers. Moreover, in that strain, a Cre-*EYK1* replicative vector can be used for transient expression of Cre recombinase [33], which faster delivers transformants without the drawbacks of using Cre-Hyg (hygromycin resistance gene) vector [23]. In summary, JMY7126 contains three auxothrophies (*Ura*-, *Lys*-, *Eyk*-), is compatible with Cre-*EYK1* (RIE132) marker rescue, and with the set of erythritol-inducible promoters deriving from *pEYK1* and *pEYD1* (Trassaert and Vandermies [19, 20], unpublished observations).

### Construction of expression vectors and CalB production strains

In most of recombinant protein production processes, protein synthesis occurs in a growth phase decoupled manner. For that purpose, promoters based on *LIP2* and *POX2* genes, strongly induced by oleic acid, have been developed (*pLIP2* and *pPOX2*) [17, 34]. The main drawback of these systems is the utilization of water-insoluble inducers, which is not convenient at large scale due to the lower mixing efficiency of industrial bioreactors. To overcome this problem, we recently developed a set of strong and tightly regulated promoters derived from *EYK1* and *EYD1* genes and induced by hydrophilic substances such as erythritol and erythrulose (Trassaert and Vandermies [19, 20], unpublished observations). Some of these promoters, namely *pEYK1*-*3AB*, *pHU8EYK*, and *pEYD1*, were selected for this study based on previous experiments (Trassaert and Vandermies [19, 20], unpublished observations) (Fig. 2).

The industrially relevant lipase CalB from *C. antarctica* was used as a model protein to assess the ability of these promoters to drive protein production in process conditions (i.e. in bioreactor). For that purpose, the CalB gene sequence, together with its pro-region was codon-optimized and fused with the signal peptide of *LIP2* (pre-region). With the resulting construct (pre*LIP2*-pro*CalB*-*CalB*, here after CalB, Additional file 1) different expression vectors were obtained, namely JME3739 (*pTEF*-CalB), JME4365 (*pEYK1*-*3AB*-CalB), JME4243 (*pHU8EYK*-CalB) and JME4590 (*pEYD1*-CalB) and JME3739 (*pTEF*-CalB) used for comparison. They were used to transform strain JMY7126, after *Not*I digestion and purification of the expression cassette. The resulting mono-copy strains, respectively JMY7536, JMY7539, JMY7544, and JMY7548, harbor a single copy of CalB expression cassette integrated at their zeta-docking platform. As stated above, the docking system prevents variability caused by random genomic integration, allowing here to compare the three selected erythritol/erythrulose-inducible promoters with the constitutive promoter *pTEF* used as a reference.

### Comparison of erythritol-inducible promoters

#### CalB gene expression and protein production in function of the erythritol-inducible promoters used

As a first characterization, CalB expression were determined after 24 h (i.e. at the end of the exponential growth phase) for strains JMY7536, JMY7539, JMY7544 and JMY7548 grown in YNBGE medium in 2Mag bioREACTOR. The culture medium employed here appears more industrially relevant, as compared to defined media previously used for CalB production [30] and more generally for recombinant protein production [35]. Rather than glucose, glycerol was selected as a main carbon source since it is a cheap by-product of the biodiesel industry that has been demonstrated suitable for recombinant protein production [36–43]. Yeast extract and soytone were added at a low concentration to the culture medium to enhance cell growth and protein production. Soytone, a peptone from soy origin, efficiently replaces casein tryptone in bioprocesses mandatorily devoid of components of animal origin [44–47]. Moreover, erythritol is a perfectly affordable inducer, especially when it could be obtained in a bioprocess from glycerol with a *Y. lipolytica* metabolically engineered strain [5] and used in combination with a *eyk1Δ* derivatives that are no longer able to consume it [18, 19].

**Fig. 3:**
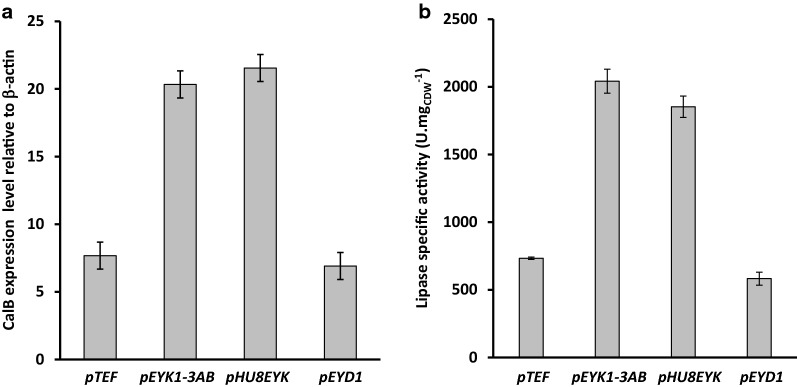
CalB gene expression level and lipase specific activity of strains JMY7536 (*pTEF*), JMY7539 (*pEYK1*-*3AB*), JMY7544 (*pHU8EYK*), and JMY7548 (*pEYD1*). Cells were grown at 28 °C in YNBGE medium in 2Mag bioREACTOR. Values are mean and standard deviation of triplicate experiments. **a** CalB gene expression level at the end of the exponential growth phase (after 24 h). Expression levels were normalized to that the one of actin. **b** Specific lipase activity after 48 h

As shown in Fig. 3a, the highest expression were obtained for *pEYK1*-*3AB* and *pHU8EYK*. Although, the CalB expression level obtained with the two promoters were not significantly different, they were, respectively, 2.5 and 2.7-fold higher than the one obtained with *pTEF* considered as a strong constitutive promoter. By contrast, *pEYD1* led to a similar expression level than the one obtained with *pTEF*.

As a further characterization, biomass and extracellular lipase activity were determined in the same experimental conditions after 48 h of growth, and the specific activities were calculated. As shown in Fig. 3b, strain JMY7539 (*pEYK1*-*3AB*) and JMY7544 (*pHU8EYK*) yielded to the highest lipase specific activities (2041 ± 78 and 1852 ± 487 U mgDCW^−1^, respectively). For those two strains, the specific enzymatic activities were, respectively, 2.8-fold and 2.5-fold higher than that obtained with strain JMY7536 (pTEF; 733 ± 88 U mgDCW^−1^). By contrast, specific lipase activity of strain JMY7548 (*pEYD1*) was 1.3-fold lower than that of strain JMY7536. The enzymatic productivit**ies** obtained here with strain JMY7539 (*pEYK1*-*3AB*) and JMY7544 (*pHU8EYK*) were 1.7 and 1.6-fold higher than the lipase productivity obtained with strain JMY1105 (*pLIP2*-*LIP2*) in 20-L batch fermentation [48]. The results obtained with strains JMY7539 (*pEYK1*-*3AB*) and JMY7544 (*pHU8EYK*) were, however, somewhat unexpected. Indeed, in previous experiments performed with fluorescent reporter system, *pHU4EYK* [19] and *pHU8EYK* (Trassaert and Vandermies, unpublished observations), bearing respectively four and eight copies of UAS1_XPR2_, significantly higher fluorescence level were obtained (4.9 and 9.8 fold, respectively) as compared to that obtained for *pEYK1*-*3AB.* However, these experiments were performed in microplate cultures that are known to be not representative of process conditions, notably in terms of oxygen transfer. Based on the results obtained for CalB gene expression and lipase specific activity, strain JMY7539 (*pEYK1*-*3AB*-*CalB*) was selected for further characterizations in batch bioreactor.

#### Culture of strain RIY368 in DASGIP bioreactor

**Fig. 4:**
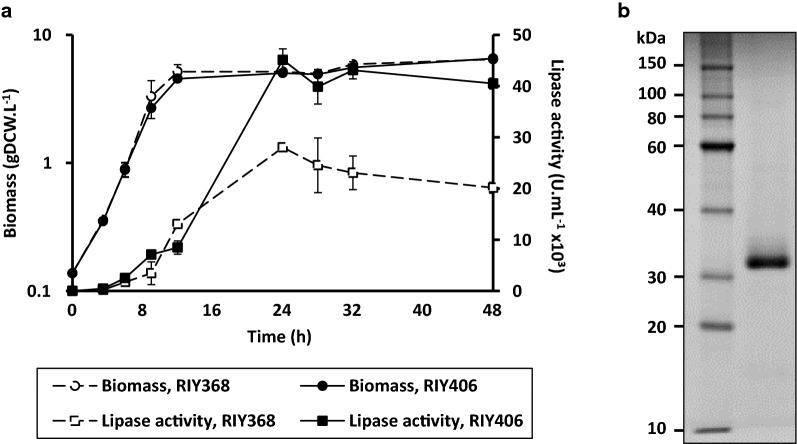
Dynamics of culture of strains RIY368 (mono-copy) and RIY406 (multi-copy) in DASGIP bioreactors. Cells were grown for 48 h at 28 °C in YNBG_2_E medium. **a** Growth curve and lipase activity of strains RIY368 and RIY406. Values are means and standard deviations of duplicate experiments. **b** SDS-PAGE gel of 5 µL of supernatant (containing 40 U of lipase CalB, sample taken at 24 h). Protein sizes are indicated on the left-hand side

To challenge the *pEYK1*-*3AB*-based expression system to process conditions, the strain RIY368, a prototroph derivative of strain JMY7539 was grown for 48 h in YNBG_2_E medium in DASGIP bioreactor, with pH and pO_2_ regulation. Samples were collected over time, and biomass together with lipase activity were determined. The exponential growth phase lasted for 12 h with specific growth rate of 0.29 ± 0.00 h^−1^ and final biomass of 6.96 ± 0.04 gDCW L^−1^ (Fig. 4, Table 3). Within the first 24 h, glycerol, the main carbon source, had been entirely consumed, and inducer (i.e. erythritol) assimilated by the cells (Additional file 2: Fig. S1). Lipase activity reached its highest titer (28,024 ± 743 U mL^−1^) after 24 h of culture (Fig. 4). It decreased slightly then after until the end of the culture (until 20150 ± 1060 U mL^−1^). Analysis of culture supernatant by SDS-PAGE clearly highlighted that CalB is the only secreted protein in those conditions (Fig. 4b). During the enzyme production phase (between 3.5 and 24 h of culture), the lipase volumetric productivity was of 1357 ± 34 U mL^−1^ h^−1^ (Table 3). Culture of strain RIY394 (*pTEF*-CalB prototroph) in the same experimental conditions yielded to a 6.2 fold lower lipase activity after 24 h (data not shown). Here, we demonstrated that a high CalB production level could be achieved in bioreactor by a combined strategy of codon optimization, and suitable inducible promoter and host strain use. In a previous bioreactor study, the native CalB sequence under the control of promoter *POX2* had been cloned in the *Y. lipolytica* strain JMY1212 (parent strain of JMY7126). The resulting lipase activity was about 5 U mL^−1^ after 102 h of cultivation [30]. Other studies in bioreactor using *K. phaffii* as a host strain for CalB production under the control of methanol-inducible promoter *pAOX1* yielded 83 U mL^−1^ in 68 h, without codon optimization [49], and 6100 U mL^−1^ in 110 h with codon optimization [50]. Here, about 28,000 U mL^−1^ of lipase CalB were obtained in 24 h. To our knowledge, this process is the most efficient identified for CalB production in yeast, and it can be safely hypothesized that such efficiency will be reflected in the production of other recombinant proteins.

**Table 3:** Dynamics of CalB production for DASGIP bioreactor cultures of strains RIY368 (mono-copy) and RIY406 (multi-copy)

Parameters	Strains	
	RIY368	RIY406
Specific cell growth rate (h^−1^)	0.29 ± 0.00	0.27 ± 0.01
Specific glycerol uptake rate (g gDCW^−1^ h^−1^)	0.37 ± 0.06	0.44 ± 0.00
Maximum lipase activity (U mL^−1^)	28,024 ± 743	45,125 ± 2144
Lipase volumetric production rate (U mL^−1^ h^−1^)	1357 ± 34	2179 ± 104

Cells were grown for 48 h at 28 °C in YNBG_2_E medium, in DASGIP bioreactors. Displayed means and standard deviations are the result of duplicate experiments

### Additional genome edition

#### Construction and screening of multi-copy CalB expressing strains

Another goal of this study was to endow the recipient strain with the possibility of additional genome editions. For that purpose an additional auxotrophy based on lysine metabolism (*lys5*) was introduced in strain JMY7126. Indeed, for some recombinant proteins, the process productivity could be enhanced by co-expression of specific chaperone as demonstrated for *K. phaffii* (see [51–53]) or by cloning an additional copy of the expression cassette [54]. To highlight this possibility of additional genome editions, strain JMY7539 (*pEYK1*-*3AB*-*CalB*) was transformed with the expression cassette of plasmid JMP4579 (*LYS5ex*-*pEYK1*-*3AB*-*CalB*, rescued beforehand by *Not*I digestion). Since the second expression cassette was integrated randomly in the yeast genome, six independent transformants were tested for their lipase activity. They were cultivated in YNBG_2_E medium in Duetz-System deepwell microplates, alongside with strain RIY368 (mono-copy). After 48 h of culture, supernatants were screened for lipase activity and it was found that the lipase productivity spanned over 3.2-fold of intensity (data not shown). The transformant presenting the highest specific lipase activity was named RIY406 and used for further experiments.

#### Culture of strain RIY406 in DASGIP bioreactor

Strain RIY406 was grown in DASGIP bioreactors under the same conditions as previously adopted for culture of mono-copy strain RIY368, in order to compare cell growth, carbon source uptake and lipase production. Cell growth kinetics of strain RIY406 was found similar to that of RIY368 (see Fig. 4 and Table 3), demonstrating that this additional recombinant gene expression did not affect cell growth capacity. Similarly to what was observed for strain RIY368, the exponential growth phase lasted for 12 h, with a specific growth rate and a final biomass of 0.27 ± 0.01 h^−1^ and 6.51 ± 0.02 gDCW L^−1^, respectively. Growth and lipase production were sustained by similar glycerol consumption in strains RIY406 and RIY368 (Additional file 2). From these data, it can be concluded that the additional metabolic load resulting from the expression of a second recombinant gene does not alter host strain metabolism. As shown in Fig. 4 and Table 3, lipase activity of RIY406 reached its highest level (45,125 ± 2144 U mL^−1^) after 24 h of culture, again after glycerol entire consumption and erythritol entire assimilation (Additional file 2). At the maximal value, RIY406 lipase activity was 1.6-fold higher than the one of RIY368. This result is consistent with the ratios of volumetric and specific production rates, which are both of 1.6 (Table 3). In conclusion, the addition of a second expression cassette based on lysine auxotrophy properly increases protein production without negative effect on the host strain. Such results confirm the opportunity of co-expression of two genes, based on two selection markers (*URA3* and *LYS5*), under the control of an erythritol/erythrulose-inducible promoter in strain JMY7126.

## Conclusions

An efficient expression system relies on four mains properties: it depends on the vector used to express the gene of interest, on the host strain used to produce the compound of interest, and on the production and downstream processing steps. The design of a given expression system arises from its use and intended versatility. Here, we developed an expression system suitable for recombinant protein production in the yeast *Y. lipolytica*. It is based on erythritol/erythrulose-inducible promoters and a dedicated host strain, JMY7126, which enables multi-copy integration of expression cassettes and prevents erythritol complete metabolization. As a proof of concept, lipase CalB was expressed in mono-copy in strain JMY7126 under the control of three types of inducible promoters. The most appropriate promoter (namely *pEYK1*-*3AB*) for the production of this given protein was selected for demonstration of multi-copy expression. Under these conditions, about 45,000 U mL^−1^ of lipase CalB were obtained in 24 h in batch bioreactor, which represents to date the most efficient process identified for CalB production in yeast. Process development shall expand the potentialities of the proposed expression system even further, and the combination may greatly improve the production of other recombinant proteins in *Y. lipolytica*.

## Supplementary information

**Additional file 1.** Nucleic and amino acid sequence of codon optimized CalB. Bold sequences correspond to the pre sequence of the extracellular lipase Lip2p encoded by the *LIP2* gene, and underlined sequences correspond to the pro CalB targeting sequence.

**Additional file 2.** Carbon source consumption of strains RIY368 (mono-copy) and RIY406 (multi-copy). Cells were grown for 48 h at 28 °C in YNBG_2_E medium, in DASGIP bioreactors. Displayed means and standard deviations are the result of duplicate experiments.

## Data Availability

Strain JMY7126 and plasmids JMP4266, JMP4230, and JME4365 are available upon MTA with INRA transfer and are patent pending.
