# Supplementary material for: Efficient expression vectors and host strain for the production of recombinant proteins by Yarrowia lipolytica in process conditions
Source: Microb Cell Fact. 2019 Oct 10;18:167. doi: 10.1186/s12934-019-1218-6 (PMC6785901; doi:10.1186/s12934-019-1218-6)
Supplement: Supplementary file 1 — Additional file 1. Nucleic and amino acid sequence of codon optimized CalB. Bold sequences correspond to the pre sequence of the extracellular lipase Lip2p encoded by the LIP2 gene, and underlined sequences correspond to the pro CalB targeting sequence. [file 12934_2019_1218_MOESM1_ESM.docx]

**ATGAAGCTTTCCACCATCCTTTTCACAGCCTGCGCTACCCTGGCTGCCGCCCTCCCTTCCCCC**ACCCCTCTGGTGAAGCGACTGCCTTCTGGATCTGACCCTGCCTTCTCTCAGCCCAAGTCTGTTCTGGACGCTGGTCTGACCTGTCAGGGAGCTTCTCCTTCTTCTGTGTCTAAGCCCATTCTCCTGGTGCCTGGAACCGGAACCACCGGTCCTCAGTCTTTCGACTCGAACTGGATTCCTCTGTCTACCCAGCTGGGATACACCCCCTGTTGGATTTCTCCTCCTCCTTTCATGCTGAACGACACCCAGGTGAACACCGAGTACATGGTGAACGCCATTACCGCTCTGTACGCTGGCTCTGGAAACAACAAGCTGCCCGTTCTGACCTGGTCTCAGGGAGGTCTGGTGGCTCAGTGGGGTCTGACCTTCTTCCCTTCTATTCGATCTAAGGTGGACCGACTGATGGCCTTCGCTCCCGACTACAAGGGAACCGTTCTGGCTGGTCCTCTGGACGCTCTGGCTGTCTCTGCTCCTTCTGTGTGGCAGCAGACCACCGGCTCTGCTCTGACCACCGCTCTGCGAAACGCTGGAGGTCTGACCCAGATTGTCCCCACCACCAACCTGTACTCTGCCACCGACGAGATTGTCCAGCCTCAGGTGTCTAACTCTCCTCTGGACTCTTCGTACCTGTTCAACGGAAAGAACATTCAGGCTCAGGCTGTCTGTGGACCTCTGTTCGACATTGACCACGCTGGCTCTCTGACCTCTCAGTTCTCCTACGTGGTTGGACGATCTGCTCTGCGATCTACCACCGGTCAGGCTCGATCTGCTGACTACGGTATCACCGACTGTAACCCTCTGCCTGCCAACGACCTGACCCCTGAGCAGAAGGTGGCTGCTGCTGCTCTGCTGGCTCCCGAGGCTGCTGCCATTGTCGCTGGTCCCAAGCAGAACTGCGAGCCCGACCTGATGCCTTACGCTCGACCCTTCGCTGTTGGAAAGCGAACCTGTTCTGGTATTGTCACCCCTTAA

**MKLSTILFTACATLAAALPSP**TPLVKRLPSGSDPAFSQPKSVLDAGLTCQGASPSSVSKPILLVPGTG**T**TGPQSFDSNWIPLSTQLGYTPCWISPPPFMLNDTQVNTEYMVNAITALYAGSGNNKLPVLTWSQGGLVAQWGLTFFPSIRSKVDRLMAFAPDYKGTVLAGPLDALAVSAPSVWQQTTGSALTTALRNAGGLTQIVPTTNLYSATDEIVQPQVSNSPLDSSYLFNGKNIQAQAVCGPLFDIDHAGSLTSQFSYVVGRSALRSTTGQARSADYGITDCNPLPANDLTPEQKVAAAALLAPEAAAIVAGPKQNCEPDLMPYARPFAVGKRTCSGIVTP*
