# Supplementary figures and images for: Efficient expression vectors and host strain for the production of recombinant proteins by Yarrowia lipolytica in process conditions
Source: Microb Cell Fact. 2019 Oct 10;18:167. doi: 10.1186/s12934-019-1218-6 (PMC6785901; doi:10.1186/s12934-019-1218-6)

## Slide 1
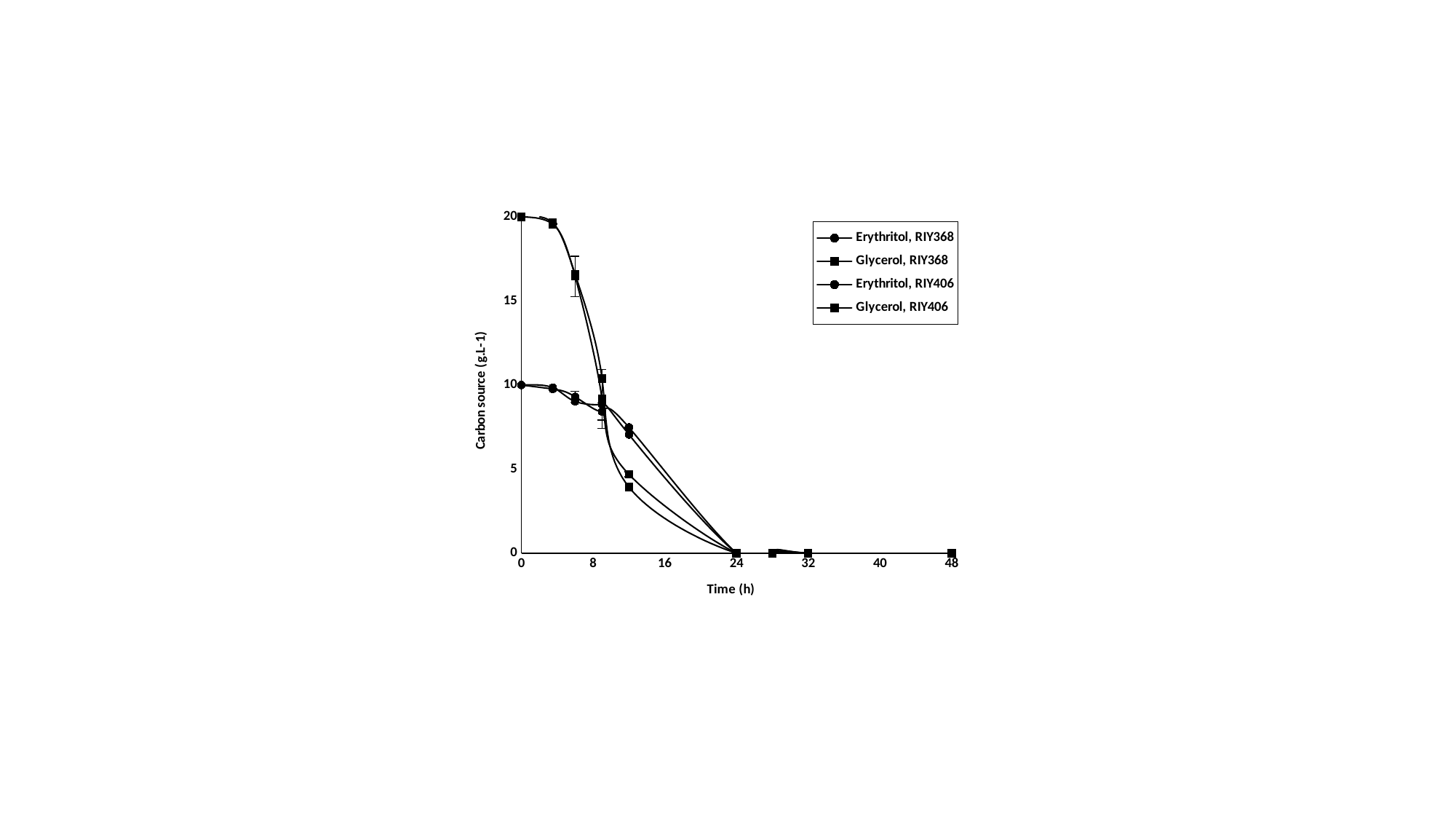

### Chart
| Category | | | | |
|---|---|---|---|---|

Supplement: Supplementary file 2 — Additional file 2. Carbon source consumption of strains RIY368 (mono-copy) and RIY406 (multi-copy). Cells were grown for 48 h at 28 °C in YNBG2E medium, in DASGIP bioreactors. Displayed means and standard deviations are the result of duplicate experiments. [file 12934_2019_1218_MOESM2_ESM.pptx]
